# Elevated aminopeptidase N affects sperm motility and early embryo development

**DOI:** 10.1371/journal.pone.0184294

**Published:** 2017-08-31

**Authors:** Amena Khatun, Md Saidur Rahman, Do-Yeal Ryu, Woo-Sung Kwon, Myung-Geol Pang

**Affiliations:** Department of Animal Science and Technology, Chung-Ang University, Anseong, Republic of Korea; National Cancer Institute, UNITED STATES

## Abstract

Aminopeptidase N (APN) is a naturally occurring ectopeptidase present in mammalian semen. Previous studies have demonstrated that APN adversely affects male fertility through the alteration of sperm motility. This enzyme constitutes 0.5 to 1% of the seminal plasma proteins, which can be transferred from the prostasomes to sperms by a fusion process. In the present study, we investigated the molecular mechanism of action of APN and its role in regulating sperm functions and male fertility. In this *in vitro* study, epididymal mouse spermatozoa were incubated in a capacitating media (pH 7) containing 20 ng/mL of recombinant mouse APN for 90 min. Our results demonstrated that the supplementation of recombinant APN in sperm culture medium significantly increased APN activity, and subsequently altered motility, hyperactivated motility, rapid and medium swimming speeds, viability, and the acrosome reaction of mouse spermatozoa. These effects were potentially caused by increased toxicity in the spermatozoa. Further, altered APN activity in sperm culture medium affected early embryonic development. Interestingly, the effect of elevated APN activity in sperm culture medium was independent of protein tyrosine phosphorylation and protein kinase A activity. On the basis of these results, we concluded that APN plays a significant role in the regulation of several sperm functions and early embryonic development. In addition, increased APN activity could potentially lead to several adverse consequences related to male fertility.

## Introduction

Mammalian spermatozoa start their challenging journey after ejaculation that lasts until they reach the oocyte. Female fallopian tubes provide a hostile environment to facilitate selection of the best spermatozoon for fertilization. During this time, each spermatozoon tries to make itself capable of fertilization through acquisition of progressive motility, hyperactivation, capacitation, and the acrosome reaction [1, 2]. In a mixed sperm population, only the progressively motile and hyperactivated spermatozoa are capable of continuing their journey until they reach the oocyte [3]. Therefore, sperm motility has been considered as one of the major factors governing male fertility [4]. Subsequently, progressively motile spermatozoa undergo capacitation via the removal of outer glycoprotein layer together with an alteration of the acrosomal cap, commonly known as the acrosome reaction. This allows a spermatozoon to attach and fuse with the oocyte during fertilization [5, 6, 7]. Alterations in membrane fluidity, protein tyrosine phosphorylation, and protein kinase A (PKA) activity are the other prime events that occur during this process [1, 8]. In particular, it has been demonstrated that seminal plasma proteins and enzymes play significant roles in controlling sperm motility, hyperactivity, and the acrosome reaction for successful fertilization [9]. Evidence showed that several seminal plasma proteins are commonly found in other cell types, such as brain, liver, blood cells, and, most relevantly, in spermatozoa [10, 11]. Among these, aminopeptidase N (APN) is a seminal plasma enzyme, commonly found in different cell types in the human body, and is associated with various disease conditions such as cancer, neoplasm, hypertension, obesity, and inflammation [12]. Interestingly, APN in human seminal plasma showed 10 to 20 times higher activity than that in the brain cells [13]. Arienti *et al*. [14] reported that APN transfers from the seminal plasma vesicles to the sperm membrane via a pH-dependent (pH ~7) fusion mechanism. Consistent with the findings of Arienti *et al*., localization of this enzyme has also been confirmed in human sperm membrane by another research group [15]. Although a number of observations made in the laboratory and during human clinical studies have suggested a possible role for altered APN levels in several diseases [16, 17], the underlying role of this enzyme in regulating male fertility remains poorly understood.

A review of literature revealed that semen from subfertile males shows relatively higher APN activity than that from their fertile counterparts [13]. A positive correlation also has been reported between the percentage of dead spermatozoa and APN activity in both the soluble and particulate sperm fractions [13]. Similarly, Subiran *et al*. [18] reported that complete inhibition of APN in mice spermatozoa resulted in increased sperm motility. In addition, a significant positive correlation has also been reported between APN activity and the number of spermatozoa with abnormal apical ridges and overall sperm defects. Although these observations provided initial insights, the specific role of this enzyme in regulating sperm functions related with male fertility, such as motility, capacitation, the acrosome reaction, protein tyrosine phosphorylation, and PKA activity, has not been investigated. To address these fundamental questions, we aimed at evaluating the effects of adding recombinant APN to sperm culture medium (capacitating media, pH 7) on sperm functions, fertilization, and early embryonic development.

## Materials and methods

### Ethical approval

All procedures were approved by the Institutional Animal Care and Use Committee (IACUC) of Chung-Ang University, Seoul, Korea. Experiments were performed according to IACUC guidelines for the ethical treatment of animals.

### Media and chemicals

All chemicals and reagents were purchased from Sigma–Aldrich (St. Louis, MO, USA), unless otherwise stated. Recombinant mouse APN was obtained from R&D Systems (Minneapolis, MN, USA). The anti-APN polyclonal antibody (CD13) was obtained from Abcam (Cambridge, UK). Modified Tyrode's medium, consisting of NaCl (97.84 mM), KCl (1.42 mM), MgCl_2_.H_2_O (0.47 mM), NaH_2_PO_4_.H_2_O (0.36 mM), D-glucose (5.56 mM), NaHCO_3_ (25 mM), CaCl_2_.H_2_O (1.78 mM), Na-lactate (24.9 mM), and gentamycin (50 μg/mL), was used as the basic medium (BM) that was supplemented with 0.4% bovine serum albumin (BSA) for inducing capacitation [19]. The pH of medium was maintained at 7 because transfer of APN from the seminal plasma vesicles to the sperm membrane is known to be activated at this pH [14]. After preincubation of spermatozoa, optimization of the initial incubation time (ranging from 30 to 90 min) and dose-dependence (ranging from 2 to 200 ng/mL) was conducted to understand the effects of APN on sperm motility. This experiment allowed us to select an optimum incubation time and dose of APN (S1 Fig). The range for dose selection was considered on the basis of the study by Huang *et al*. [20].

### Collection and preparation of mouse spermatozoa

One day before the start of the experiment, BM was incubated with additional APN (20 ng/mL) at 37°C in an atmosphere of 5% CO_2_. Mouse spermatozoa were collected from the cauda epididymides of 12-week-old ICR mice (Nara Biotech, Seoul, Korea), as described by Lee *et al*. [19]. Briefly, the cauda epididymides were collected and transferred to cell culture dishes containing 2 mL of BM. After a 12-min preincubation, spermatozoa were incubated in BM media with 0.4% BSA for inducing capacitation [19, 21] (with/without APN) for 90 min at 7°C in an atmosphere of 5% CO_2_. For each experiment, three male mice per replicate were used.

### Immunofluorescence assay

The subcellular localization of proteins is very important because it can be readily used to obtain information about their potential function. To evaluate the subcellular localization of APN in the freshly collected epididymal mouse spermatozoa, an immunofluorescence assay was performed using the CD13 antibody. Briefly, the air-dried spermatozoa placed on glass slides were fixed with 3.7% paraformaldehyde for 30 min at 4°C [22]. After washing with Dulbecco's phosphate-buffered saline (DPBS) containing 0.1% Tween 20 (PBS-T) and blocking for 1 h in the blocking solution (5% BSA in PBS-T) at 4°C, the slides were incubated with diluted rabbit polyclonal primary antibody for APN (1:100; Abcam) in blocking solution, and diluted lectin PNA 34 and 35 antibodies (1:100) conjugated with Alexa Fluor 647 (Molecular Probes) in blocking solution overnight at 4°C. After washing, the slides were incubated for 2 h at room temperature (RT) with diluted fluorescein isothiocyanate-conjugated goat polyclonal secondary antibody to rabbit IgG (1:100; Abcam) in blocking solution. After applying the Hoechst 33342 and antifade reagents, the samples were observed with a Nikon TS-1000 microscope using the NIS-Elements imaging software (Nikon, Tokyo, Japan).

### Computer-assisted sperm analysis (CASA)

Briefly, after collecting the epidydimal spermatozoa, they were incubated for 90 min in the capacitating media with/without APN, and the sperm motility (%), velocity distribution, and kinematic parameters [hyperactive motility (HYP%), curvilinear velocity (VCL), velocity straight line (VSL), velocity average path (VAP), linearity (LIN%), path straightness (STR), beat cross-frequency (BCF), mean angular displacement (MAD), wobble coefficient (WOB), dance mean (DNM), and lateral head displacement (ALH)] were measured using the CASA system (SAIS Plus version 10.1; Medical Supply, Seoul, Korea). The phase contrast objective (magnification, 10 ×) was used with the SAIS software to analyze the spermatozoa. Sperm motion kinematics were measured, as described previously [19].

### Measurement of enzyme activity

APN activity of the sperm culture medium was determined according to the method described by Viudes de Castro *et al*. [23]. Briefly, APN activity was monitored using a commercially available substrate, namely H-Ala-7-amino-4-methylcoumarin (Bachem, Bubendorf, Switzerland). Briefly, the samples (50 μL) were incubated with the above substrate in assay buffer for 30 min at 37°C, after which, the reaction was stopped by adding 0.1 M sodium acetate buffer (pH 4.2). Cellular APN activity was monitored in terms of the amount of 7-amino-4-methyl-coumarin (AMC) released, measured by using a microplate fluorometer, with maximum excitation and emission wavelengths of 380 and 460 nm, respectively, and the SoftMax Pro 5 software (Molecular Devices, Sunnyvale, CA, USA). It has been reported that the fluorescence intensity is proportional to the level of APN activity.

### Hypo-osmotic swelling test (HOST)

First, 100 μL of the control and treated samples were gently mixed with 900 μL of the hypo-osmotic solution (distilled water:NaCl (0.9%), 1:1; 150 mOsm/kg) individually and incubated for 30 min at 37°C in an atmosphere of 5% CO_2_ in air. After incubation, 50 μL of the sample solution was spread over a glass slide, allowed to air dry, and fixed with fresh fixative (methanol:glacial acetic acid, 3:1). For each treatment, the number of viable cells were counted using the Microphot-FXA microscope (Nikon). At least 500 cells were evaluated per slide.

### Combined Hoechst 33258/chlortetracycline (CTC) fluorescence assessment of spermatozoa

To determine the capacitation status of the spermatozoa from both the control and treated sperm culture media, CTC staining assays were performed as described previously [21]. After treatment, 15 μL of Hoechst33258 solution was added to 135 μL of the sperm sample and incubated for 2 min at RT. Next, 250 μL of 2% polyvinylpyrrolidone in DPBS was added and centrifuged at 100 *g* for 2.5 min. Cell pellets were resuspended in 100 μL of DPBS and 100 μL of CTC solution, as described previously [19]. The capacitation status was determined using the Microphot-FXA microscope (Nikon) under epifluorescence illumination, using the ultraviolet BP 340–380/LP 425 and BP 450–490/LP 515 excitation/emission filters for H33258 and CTC, respectively. As per the published criteria [24], this analysis revealed four different patterns indicating different capacitation status: dead, blue fluorescence pattern over the head (D); live noncapacitated, bright green fluorescence pattern over the entire sperm head (F); live capacitated, bright green fluorescence pattern over the acrosomal region with a dark post-acrosomal region (B); or live acrosome-reacted, with no fluorescence over the head (AR). At least 400 spermatozoa were evaluated per treatment slide.

### Detection of lactate dehydrogenase (LDH)

To determine cellular cytotoxicity, we used the CytoTox 96^®^ Nonradioactive Cytotoxicity assay kit (Promega, Fitchburg, WI, USA), which is based on the calorimetric detection of released LDH. After collection of the epidydimal spermatozoa, they were incubated for 90 min in the capacitating medium with/without APN. Next, 20 μL of lysis buffer was added to 200 μL of both the control and treated sperm groups and incubated at 37°C for 1 h. Subsequently, the supernatant was collected after centrifugation at 250 *g* for 4 min. For each treatment, 50 μL of substrate was added to 50 μL of supernatant in a 96-well plate and incubated in dark at RT for 30 min. LDH activity was measured as the absorbance at 490 nm using a luminometer (Gemini Em; Molecular Devices) and calculated using the SoftMax Pro 5 software. LDH activity was reported as the absorbance value for each tested sperm group, as described previously [21].

### Measurement of cellular reactive oxygen species (ROS)

Cellular ROS were measured using the oxidation-sensitive fluorescent dye 2′,7′-dichlorofluorescein diacetate (DCFDA), 1 × buffer, and 1 × supplemental buffer (Abcam) according to the manufacturer's instructions and a previously described method [25]. After collection of the epidydimal spermatozoa, they were incubated for 90 min in the capacitating medium with/without APN. The samples were washed at 300 *g* for 4 min and resuspended in 1 mL of the DCFDA mix. After incubating for 30 min at 37°C, the samples were washed with 1 × buffer solution at 300 *g* for 4 min, and resuspended in 500 μL of 1 × supplemental buffer. Finally, the cell suspension (500 μL) was placed in a 96-well plate. Each sperm suspension was exposed to an excitation wavelength of 485 nm, and subsequently, the emitted fluorescence was measured at 535 nm. Fluorescence was detected using a microplate fluorometer (Gemini Em) and calculated using the SoftMax Pro 5 software (Molecular Devices). The fluorescence values represented a measure of the activity of ROS in each group.

### Western blot analysis

Western blotting was performed for the detection of APN, phospho-PKA substrate, and tyrosine-phosphorylated proteins in spermatozoa, as described previously [21]. Briefly, after collection of the epidydimal spermatozoa, they were incubated for 90 min in the capacitating medium with/without APN. All samples were washed three times with DPBS by centrifugation at 10,000 *g* for 5 min. The supernatants were discarded and sperm pellets were resuspended in the Laemmli sample buffer (63 mM Tris, 10% glycerol, 10% sodium dodecyl sulfate, and 5% bromophenol blue) containing 2-mercaptoethanol (5%) and incubated at RT for 10 min. Next, the supernatants were collected by centrifugation at 10,000 rpm for 10 min and boiled at 100°C for 3 min. The proteins were resolved on 12% SDS-PAGE using a mini-gel system (Amersham, Piscataway, NJ, USA). The separated proteins were transferred onto polyvinylidene fluoride membranes (Amersham). Subsequently, the membranes were blocked at RT for 2 h with the blocking solution. They were washed twice for 2 min with PBS-T. To detect APN, the membrane was incubated overnight with the rabbit polyclonal primary antibody for APN (1:1000; Abcam) at 4°C. After washing with PBS-T, the membrane was incubated with the horseradish peroxidase (HRP)-conjugated goat anti-rabbit IgG secondary antibody (1:3000; Abcam) at RT for 2 h. For detection of the phospho-PKA substrates, the membrane was incubated with the anti-phospho-PKA substrate rabbit monoclonal antibody (1:5000; Cell Signaling Technology, Danvers, MA, USA) overnight at 4°C. Tyrosine phosphorylation was detected using the HRP-conjugated mouse monoclonal anti-phosphotyrosine antibody (PY20, 1:2500; Abcam) overnight at 4°C. As an internal control, α-tubulin was detected using the anti-α-tubulin mouse antibody (1:5000; Abcam). Detection was performed by the enhanced chemiluminescence (ECL) technique using ECL reagents. Bands were scanned and visualized using the GS-800 Calibrated Imaging Densitometer (Bio-Rad, Hercules, CA, USA) and analyzed using the Quantity One software (Bio-Rad). For each sample, quantification of bands was performed by determining the ratios of APN, phospho-PKA substrates, and tyrosine-phosphorylated proteins to α-tubulin.

### *In vitro* fertilization (IVF)

To investigate the effect of high APN activity on fertilization and embryo development, 12-week-old female B6D2F1/CrljOri hybrid mice were purchased from Nara Biotech. These mice were superovulated with 5 IU of pregnant mare serum gonadotropin and 5 IU of human chorionic gonadotropin by intraperitoneal (ip) injections, separated by an interval of 48 h. Fifteen hours after the second injection, the cumulus-oocyte complexes (COCs) were collected and transferred to BM supplemented with 10% fetal bovine serum (FBS) under mineral oil and incubated for 1 h at 37°C in an atmosphere of 5% CO_2_ in air. To rule out the sex-specific (female) factors and individual variations, the collected oocytes from one mice were divided equally for the *in vitro* fertilization assay. We used about 30 oocytes for each trial. After collection of the epidydimal spermatozoa, they were incubated for 90 min in the capacitating medium with/without APN. After inducing capacitation, both control and treated spermatozoa were washed with 0.4% BSA-containing BM, and 1 × 10^6^/mL spermatozoa were gently inseminated into the incubated oocytes. The oocytes were incubated for 6 h at 37°C in an atmosphere of 5% CO_2_ in air, as described previously [21]. After fertilization, the normal embryos were transferred to fresh 0.4% BSA-containing BM (50 μL) and incubated. Fertilization rate was evaluated by counting the two-cell embryos 24-h post-insemination. All the two-cell embryos were transferred to fresh 0.4% BSA-containing BM for 5 days at 37°C in an atmosphere of 5% CO_2_ in air. After 5 days, the number of blastocysts were counted.

### Bioinformatic analysis

To investigate the interacting proteins, cellular regulation checkpoints, and diseases related to APN, we used the Pathway Studio program (Elsevier, Amsterdam, The Netherlands). This program allowed us to search for the molecular interactions after selecting APN as the input object. Information retrieved from the program was reconfirmed by checking the PubMed Medline hyperlink that was embedded in each node.

### Statistical analyses

Data were assessed for normal distribution using the Shapiro–Wilk test. The data showing normal distribution were further analyzed using the two-tailed Student’s *t*-test. The data showing nonparametric patterns were analyzed using the Mann–Whitney U test, to analyze significant differences. All analyses were performed using the SPSS statistical software (version 12.0; Chicago, IL, USA) For a significant difference to be observed, a consistent and reasonable difference in magnitude is required between the control and treated samples; *P* < 0.05 was considered to be statistically significant. Numerical data have been represented as the mean ± standard error of the mean (SEM).

## Results

### Immunolocalization of APN in the post-acrosomal region of spermatozoa

**Fig 1:**
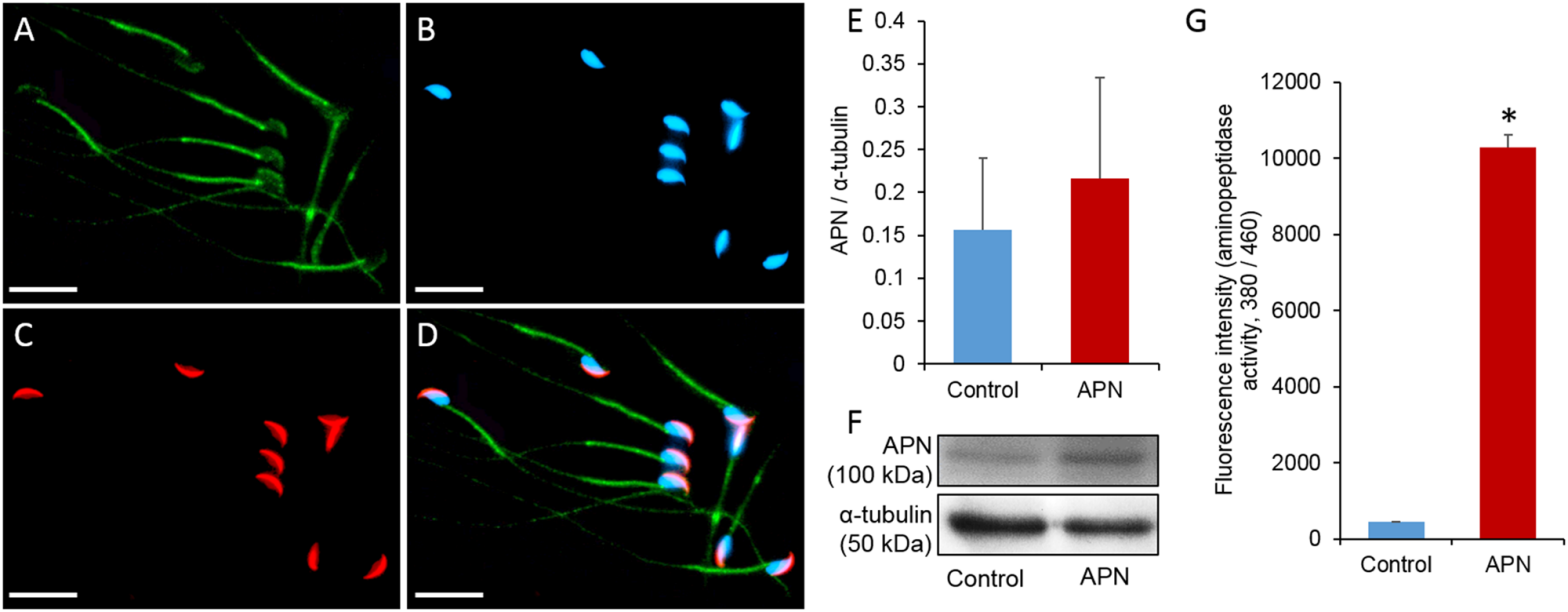
Localization of APN in spermatozoa and the effect of recombinant APN (20 ng/mL) on APN level and its enzymatic activity in mouse spermatozoa. (A) Localization of APN (green). (B) Location of nucleus (Hoechst 33258, blue). (C) Location of the acrosome (lectin PNA, red). (D) Merged location of the nucleus, acrosome, and APN. Images were obtained using the Nikon TS-1000 microscope and NIS-Elements imaging software (Nikon, Japan). Bar = 20 μm. (E) Quantification of APN in spermatozoa. (F) Representative image of western blot showing the band (~100 kDa) corresponding to APN in the spermatozoa (*P* = 0.234). (G) Enzymatic activity of APN in the control and treated groups (*P* = 0.00001). Data represent the mean ± SEM of three replicates. *P < 0.05, calculated using two-tailed Student’s *t*-test.

We evaluated the immunolocalization of APN in mice spermatozoa. Fig 1 shows that APN was localized in the post-acrosomal region of the sperm head, over the midpiece, and in the tail.

### Addition of recombinant APN increases APN activity in sperm culture medium

We evaluated APN activity and protein levels in spermatozoa following the addition of APN to the sperm culture medium. Data showed that the addition of 20 ng/mL of recombinant APN significantly increased the APN activity in spermatozoa (*P* = 0.00001) compared to the control group. A non-significant difference was observed in the levels of APN protein between the treated and control groups (*P* = 0.234) (Fig 1).

### Increased APN activity affects motility of spermatozoa

**Fig 2:**
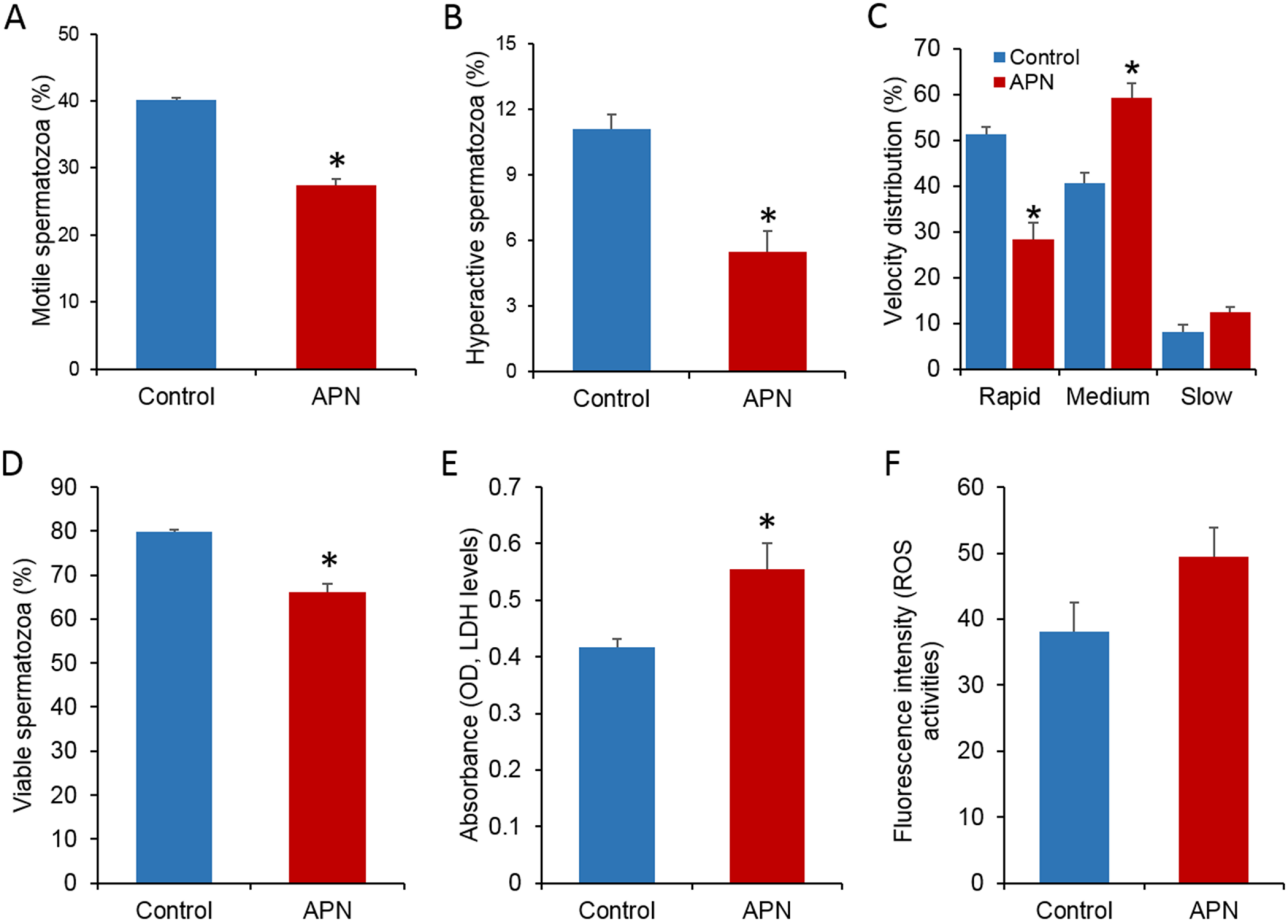
Effects of addition of recombinant APN (20 ng/mL) on motility, hyperactive motility, viability, LDH, and intracellular ROS levels in mice spermatozoa. (A) Sperm motility (%) (*P* = 0.00001). (B) Hyperactive motility (HYP%) (*P* = 0.003). Data represent the mean ± SEM of four replicates. (C) The percentage of rapid- (*P* = 0.00001), medium- (*P* = 0.001), and slow-speed (*P* = 0.053) spermatozoa (D) The percentage of viable spermatozoa (*P* = 0.002). (E) LDH levels (*P* = 0.045). (F) Intracellular ROS levels (*P* = 0.142). Data represent the mean ± SEM of three replicates. *P < 0.05, calculated using two-tailed Student’s *t*-test.

We also evaluated the effect of increased APN activity in the sperm culture medium on sperm motility and motion kinematics. As shown in Fig 2, the percentage of motile (*P* = 0.00001), hyperactivated motile (*P* = 0.003), and rapid-speed (*P* = 0.00013) spermatozoa was significantly lower in the treated group compared to the control group. In contrast, the percentage of medium-speed spermatozoa was significantly higher in the treated group (*P* = 0.001) compared to the control group. Interestingly, no significant difference was observed among the percentage of slow-speed spermatozoa between the two groups (*P* = 0.053). Other parameters, namely VCL, VSL, VAP, LIN, STR, WOB, ALH, and BCF, were not affected (data not shown). The average VAP and STR values for spermatozoa were less than 50 μm/s and 80, respectively, in the APN-supplemented sperm culture medium. In contrast, the average VAP and STR values for spermatozoa were more than 50 μm/s and 80, respectively, in the sperm culture medium without APN (S1 Table).

### APN supplementation affects cell viability and LDH level, but not ROS level

To investigate the effects of recombinant APN supplementation in sperm culture medium on sperm viability, cytotoxicity, and ROS levels, HOST, LDH measurement, and fluorometric assays were performed, respectively. The results showed that the percentage of viable spermatozoa decreased significantly in the presence of high APN activity in the sperm culture medium (*P* = 0.002), correspondent with increased LDH levels (*P* = 0.045). However, no significant difference in ROS level was observed between the control and treated spermatozoa (Fig 2).

### Increased APN activity does not affect the capacitation status, protein tyrosine phosphorylation, and PKA activity in spermatozoa

**Fig 3:**
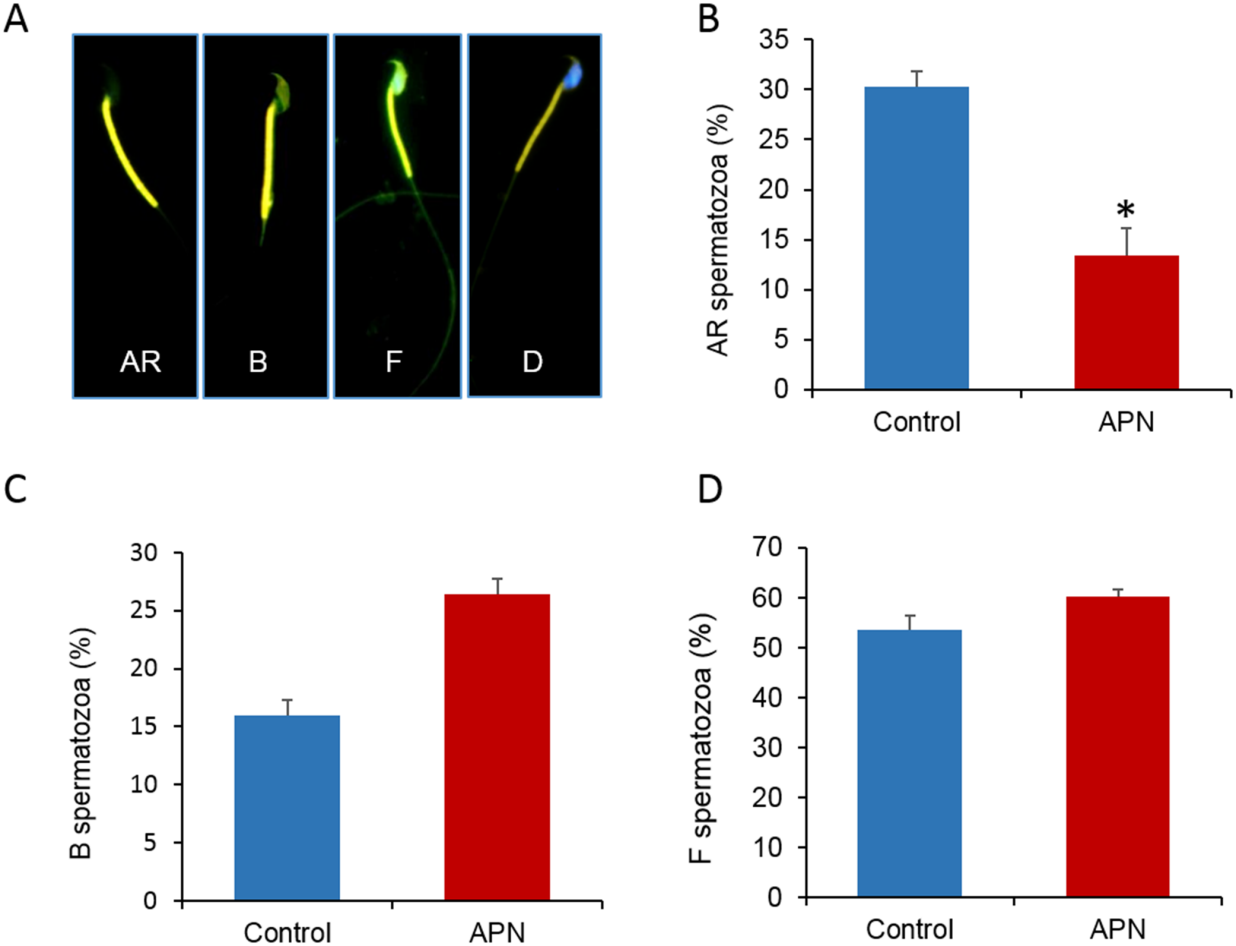
Effect of recombinant APN (20 ng/mL) on the capacitation status of mouse spermatozoa. Different patterns of spermatozoa, such as (A) acrosome-reacted (AR pattern), capacitated (B pattern), non capacitated (F pattern), and dead (D pattern), observed after combined Hoechst 33258/chlortetracycline fluorescence staining. (B) Difference in AR pattern spermatozoa (*P* = 0.006). (C) Difference in B pattern spermatozoa (*P* = 0.100). (D) Difference in F pattern spermatozoa (*P* = 0.123). Data represent the mean ± SEM of three replicates. *P < 0.05, calculated using two-tailed Student’s *t*-test.

**Fig 4:**
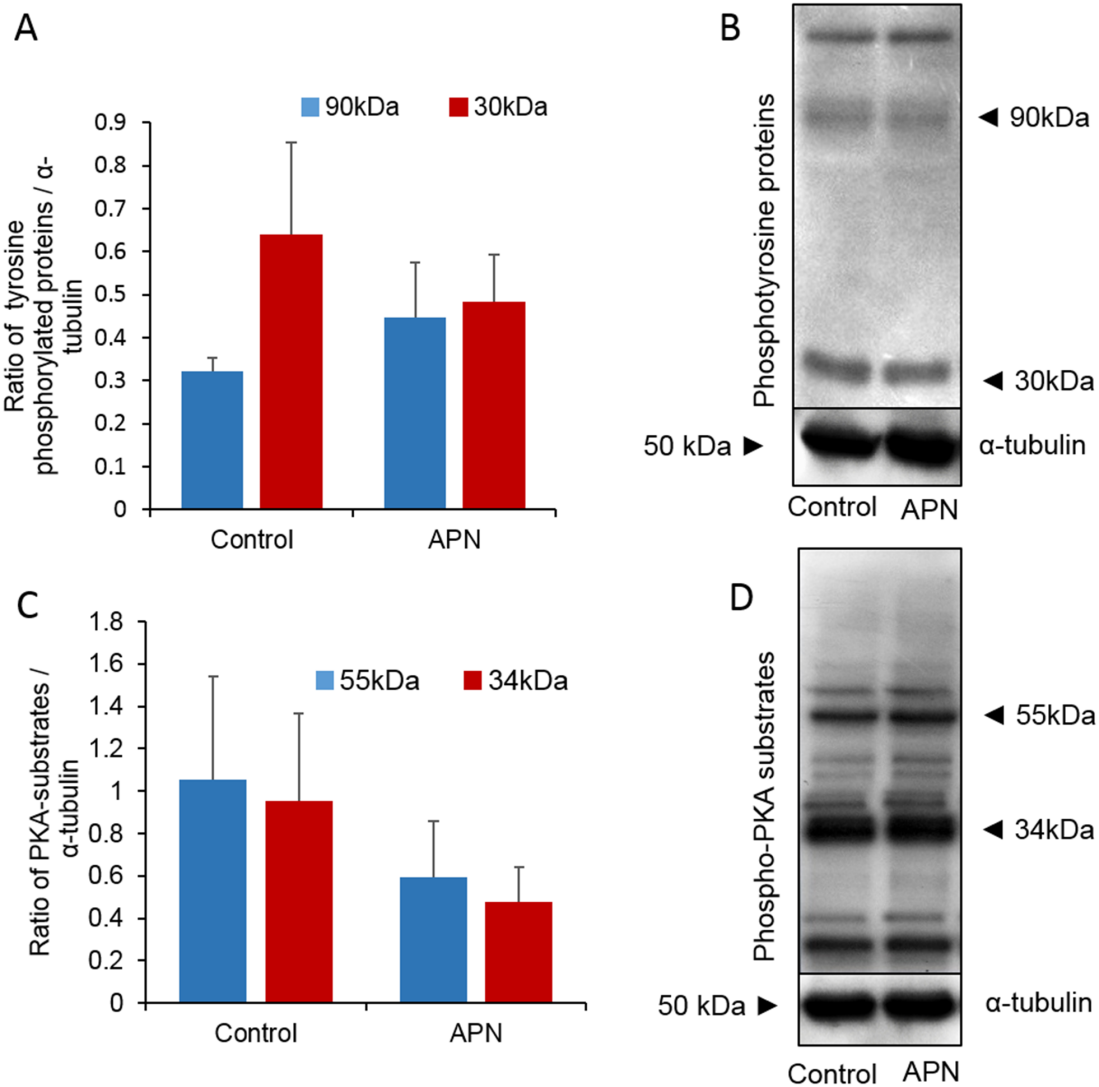
Effect of addition of recombinant APN (20 ng/mL) on protein tyrosine phosphorylation and PKA activity in mouse spermatozoa. (A) Densities of tyrosine phosphorylated proteins (~90 and 30 kDa) in the control and treated groups (*P* = 0.179 and 0.215, respectively). (B) Representative image of the western blot for tyrosine phosphorylated proteins. (C) Densities of PKA substrates (~55 and 34 kDa) in the control and treated groups (*P* = 0.353 and 0.342, respectively). (D) Representative image of the western blot for phospho-PKA substrates. Data represent the mean ± SEM of three replicates. *P < 0.05, calculated using two-tailed Student’s *t*-test.

Significantly lesser acrosome-reacted (AR pattern) spermatozoa were observed after treatment with APN (*P* = 0.006). In contrast, the number of capacitated (B pattern) spermatozoa increased substantially, but the difference was not statistically significant (*P* = 0.100) even after high APN supplementation. In case of non-normal distribution, data were analyzed for significant differences using the Mann–Whitney U test (Fig 3). Next, we evaluated protein tyrosine phosphorylation and PKA activity by western blotting. Although two different bands (~30 and 90 kDa) were detected for tyrosine phosphorylation in both the treated and control spermatozoa, the extent of tyrosine phosphorylation was not significantly different for both the bands (*P* = 0.353 and 0.342, respectively) between the two groups. Consistent with these results, the PKA activity was also not significantly different in the control and treated spermatozoa for the ~34 and 55 kDa bands (*P* = 0.179 and 0.215, respectively) (Fig 4).

### Increased APN activity does not affect fertilization, but affects early embryonic development

**Fig 5:**
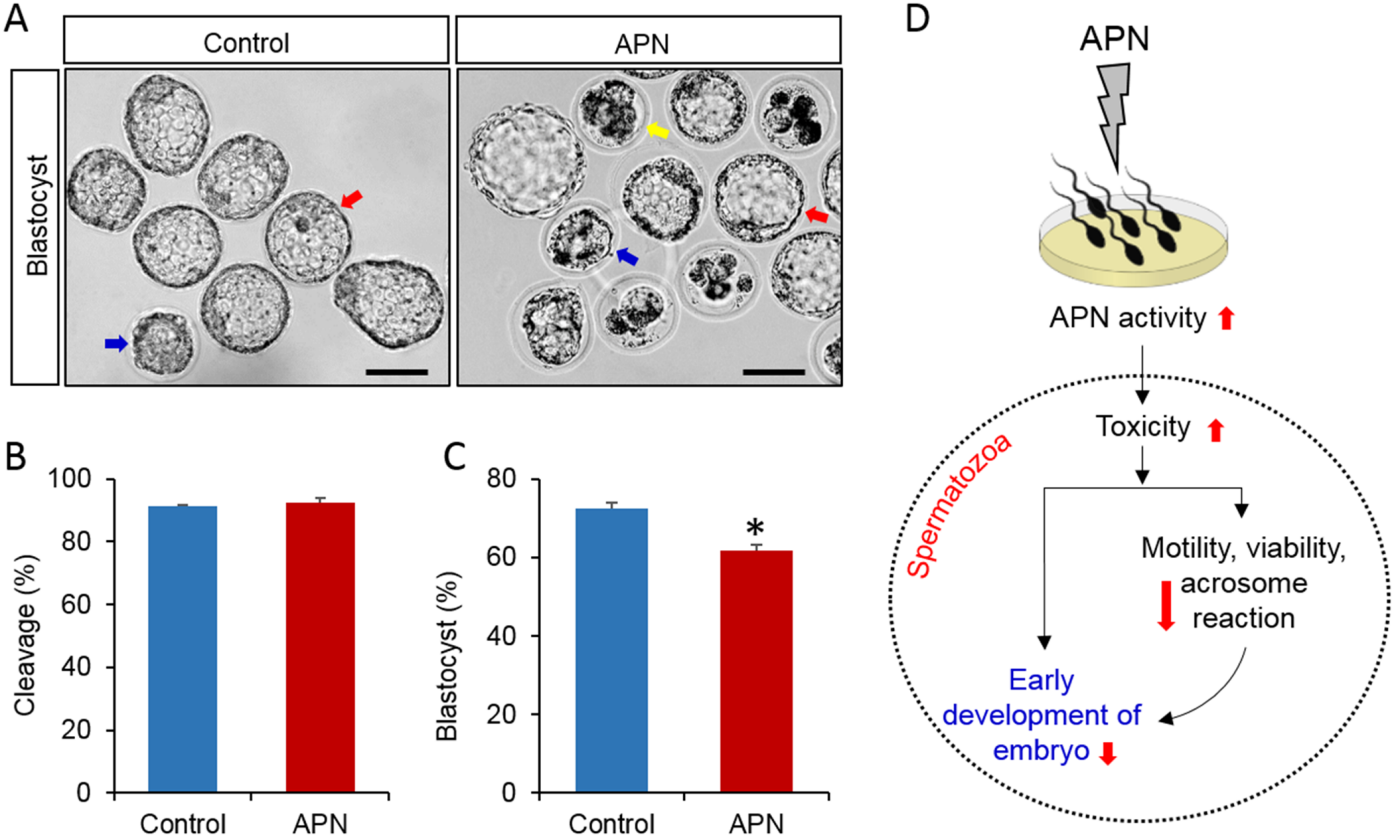
Effect of recombinant APN on fertilization and embryonic development. (A) Blastocyst quality in the control and APN-treated groups (Bar = 50 μM). Red, blue, and yellow arrows indicate grade 1, 2, and 3 blastocysts, respectively. (B) Difference in cleavage rate between the control and treated groups (*P* = 0.568). (C) Blastocyst formation rate in the control and treated groups (*P* = 0.008). Data represent the mean ± SEM of three replicates. *P < 0.05, calculated using two-tailed Student’s *t*-test. (D) Effect of APN on mouse spermatozoa and its hypothetical mechanisms of action. High APN activity induces toxicity in spermatozoa and subsequently decreases sperm motility, viability, and acrosome reaction. Ultimately, the affected spermatozoa display pathological conditions induced by dysregulation of early embryonic development.

We detected the effect of increased APN activity in sperm culture medium on fertilization and early embryonic development. Fertilization was not affected by APN treatment (*P* = 0.568). Intriguingly, a significant inhibition of embryonic development was observed for the APN-treated spermatozoa (*P* = 0.008) (Fig 5). According to blastocyst grading (1 to 3) by Balaban *et al*. [26], most blastocysts in the control group were categorized as grade 1 blastocysts, while grade 2 and grade 3 blastocysts were predominantly observed in the APN-treated group (Fig 5).

### Bioinformatic analysis using APN

**Fig 6:**
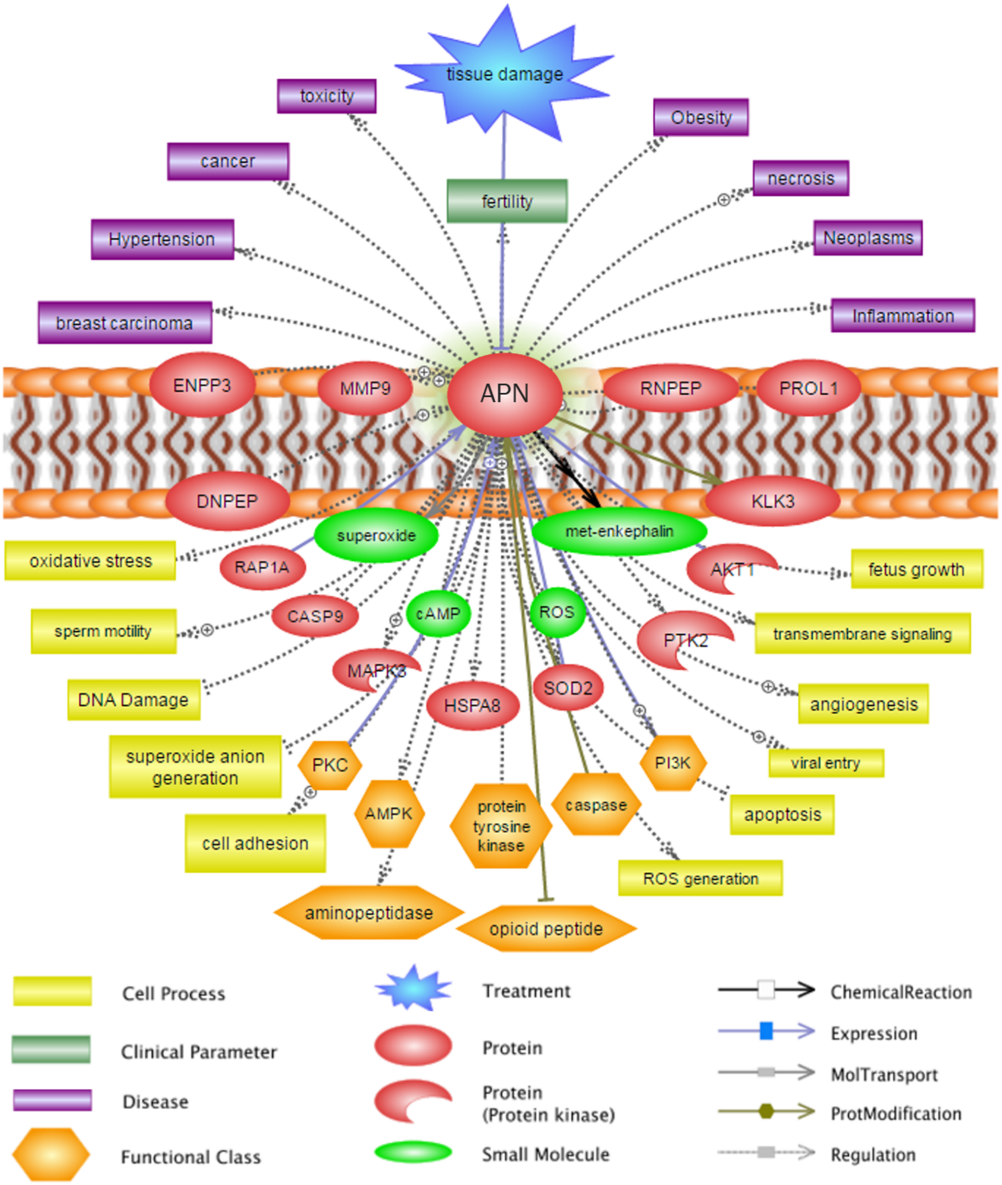
Illustration of the APN-regulated proteins, cellular functions, and diseases analyzed using the Pathway Studio program.

Although numerous roles of APN have been elucidated in many cells and organs, there is little information on its role in the sperm cells. Various studies have proposed that all cell types share a common function through their regulatory network [27]. Therefore, we used bioinformatic tools to identify the cellular functions of APN that may regulate sperm cell functioning and fertilizing potential. In the present study, investigation of protein interactions, cellular functions, and diseases related to APN was performed using the Pathway Studio program. The results showed that APN interacted with several proteins, such as protein kinases, superoxide dismutase, enkephalin, caspase, Ras-related proteins, heat shock proteins, and matrix metalloproteinase. Our analysis also revealed that these proteins potentially regulate several important cellular processes, including oxidative stress, apoptosis, angiogenesis, ROS regulation, DNA damage, and cell adhesion. Finally, functional association among these proteins was observed to be important for the pathogenesis of several conditions, such as fertility regulation, neoplasia, cancer, obesity, and inflammation (Fig 6).

## Discussion

APN is a moonlighting enzyme that regulates several important functions in the body associated with growth and development [12]. It constitutes up to 0.5 to 1% of the seminal plasma proteins [28]. It has been reported that the seminal plasma proteins play a potential role in the regulation of male fertility [29]. However, the relationship between APN and sperm/male fertility reported by several investigators remains discordant [13, 15, 17, 18]. In the present study, we supplemented APN in sperm culture medium during capacitation in order to understand the specific role of APN in the regulation of sperm function(s) and fertilization.

Sperm motility is a critical factor that provides a qualitative assessment for the prediction of semen fertility. Only progressively motile spermatozoa are able to reach the oocyte for fertilization. In the present study, sperm motility and motion kinematic parameters were investigated using CASA. We demonstrated that the addition of recombinant APN in sperm culture medium significantly inhibited the percentage of motile, hyperactivated, and rapid-speed spermatozoa during capacitation. However, the percentage of medium-speed spermatozoa was significantly higher in the APN-supplemented group (Fig 2). Based on previous studies, a progressively motile spermatozoon can be defined as a spermatozoon with > 50 μm/s VAP [30] and > 80 STR [31]. The present study demonstrated that high APN activity in sperm culture medium affected spermatozoa by decreasing their average VAP and STR (S1 Table). Thus, we concluded that the addition of APN affected the progressive motility of spermatozoa. As expected, no difference was observed in APN expression between the control and treated groups (Fig 1). This was mostly because spermatozoa are believed to be transcriptionally and translationally silent, and therefore, are incapable of protein synthesis [32]. Concurrently, high APN activity in seminal plasma has also been reported in subfertile males [13]. In addition, Subiran *et al*. [18] reported that the addition of leuhistin, a specific inhibitor of APN, could increase the motility of mice spermatozoa. Therefore, we hypothesized that higher APN activity in seminal plasma/sperm culture medium had an inhibitory effect on sperm motility.

A positive correlation between necrozoospermia and high APN activity has been reported in a previous study [13]. Piva *et al*. [33] showed increased APN activity in HeLa cells following *in vitro* exposure to chemical stress, subsequently leading to an upsurge in transforming growth factor-alpha and bioactive molecules. Nevertheless, the association between altered APN activity and germ cell death is not completely understood. To investigate the mechanisms underlying decreased sperm motility in APN-rich medium, we evaluated sperm viability and the level of cellular LDH, a cytosolic enzyme released during cell damage. As expected, the spermatozoa treated with recombinant APN showed significantly lower cell viability (Fig 2D) and higher LDH levels. Interestingly, although a slight increase in ROS level was observed following APN supplementation, the alteration was statistically insignificant. Identification of ROS using oxidized DCFDA and fluorometry might be less sensitive for detecting cellular ROS level. In order to compensate for this weakness and measure the levels of cellular ROS, the use of dichlorofluorescein diacetate (DCFH-DA) and flow cytometry is recommended [34]. It has been demonstrated that increased LDH and ROS levels may lead to male infertility by decreasing motility and hyperactivation of mice spermatozoa [35]. In this study, increased APN activity might indicate a toxic cellular microenvironment (increased LDH level) that subsequently reduced sperm motility (Fig 2E).

Following ejaculation, mammalian spermatozoa become competent to fertilize an egg through unique processes called capacitation and the acrosome reaction. The functional modifications of spermatozoa are regulated by several signaling pathways, most importantly, those involving the PKA-dependent phosphorylation of tyrosine residues [8]. In this study, we demonstrated that higher APN activity decreased the percentage of acrosome-reacted spermatozoa. Interestingly, the decrease in the acrosome reaction rate was independent of the PKA activity and protein tyrosine phosphorylation (Fig 4). In addition, we confirmed the localization of APN in the post-acrosomal, midpiece, and tail regions of mouse spermatozoa (Fig 1). It has been reported that understanding the compartmentalization of specific proteins within cells provides a basic idea of their potential cellular function(s) [36, 37]. Therefore, localization of APN in the post-acrosomal region hinted at its potential functional relationship with the acrosome reaction, while localization in the neck and tail regions may indicate its role in the regulation of sperm motility [38].

Recently, Viudes de Castro *et al*. [23] also showed a significant negative correlation between APN activity and the acrosome reaction in rabbit spermatozoa. It has been reported that the role of the seminal plasma enzyme enkephalin is important for the regulation of the sperm acrosome reaction [39]. Since APN is an enkephalin-degrading enzyme, the decrease in acrosome reaction rate observed in our present study might be regulated indirectly by the degradation of enkephalin. Bioinformatic analysis revealed that APN facilitated the chemical reaction with enkephalins (Fig 6). Consistent with the findings of previous studies, our findings suggested that APN may suppress the acrosome reaction by altering enkephalins in spermatozoa. However, further studies are required to confirm this hypothesis.

The most important result from this study was that high APN activity had a significant inhibitory effect on early embryonic development; however, it did not affect fertilization (Fig 5). An efficient sperm–egg interaction is necessary for embryo development; therefore, functional integrity of both cells is extremely important [40]. If cellular toxicity is increased, it may cause DNA damage and oxidative stress in spermatozoa, subsequently affecting its motility [41]. Moreover, the quality of sperm DNA also has a direct influence on human embryo development [42, 43, 44]. It has been reported that oocytes are capable of repairing sperm DNA damage [45]. Therefore, a spermatozoon with substantially high DNA fragmentation, can still fertilize an oocyte [45]. The repair of sperm DNA fragmentation basically depends on the cumulative effects of sperm chromatin damage and the ability of the oocyte to repair it. Gawecka *et al*. [45] also reported that the ability of oocytes to recover abnormal sperm DNA is mostly dependent on the quality of oocyte and type of DNA damage in spermatozoa. Consistent with these findings, the APN-mediated increase in cytotoxicity noticed in the present study (Fig 4) may cause persistent damage to the spermatozoa. As such, even though they were able to fertilize the oocyte (collected from wild type mice), the damaged spermatozoa were incapable of continuing embryo development any further.

To elucidate the role of APN in spermatozoa, we investigated the interactions of APN with other proteins and their regulatory mechanisms in the pathogenesis of diseases using the Pathway studio program. Proteins do not work independently. They interact with a wide range of molecules to regulate diverse cellular process. We noted that APN potentially interacts with several other proteins, most importantly protein kinases, superoxide dismutase, enkephalin, caspase, Ras-related proteins, heat shock protein, and matrix metalloproteinase. It is also important to note that most proteins that interact with APN are extremely important in the regulation of normal sperm functions and fertilization. Therefore, increased APN activity might have a significant influence on the functioning of other interacting proteins. We also illustrated (Fig 6) that functional alteration of these proteins may affect several biological processes, such as oxidative stress response, cell cycle progression, transmembrane signaling, and DNA integrity, finally resulting in increased predisposition towards pathological conditions.

To the best of our knowledge, this is the first comprehensive *in vitro* study to investigate the effect of high APN activity (in sperm culture medium) on spermatozoa. We concluded that APN plays a significant role in the regulation of several sperm functions. Further, high APN activity affected sperm motility, viability, acrosome reaction, and early embryonic development. Therefore, exposure of spermatozoa to increased APN levels may disturb cellular homeostasis, and subsequently result in several adverse consequences (Fig 5D). These effects might also be mediated by the functional alteration of other interacting proteins. However, further studies are necessary to prove this hypothesis.

## Supporting information

S1 Table Comparison of the average path velocity (VAP) and straightness (STR) between the control and APN-supplemented spermatozoa. (DOCX)

S1 Fig Effect of different concentrations (2–200 ng/mL) of recombinant APN incubated for 30–90 min on the motility of mice spermatozoa. Data represent the mean ± SEM of six replicates. *P < 0.05, calculated using Tukey’s multiple comparison test.
(DOCX)
