# Supplementary material for: Elevated aminopeptidase N affects sperm motility and early embryo development
Source: PLoS One. 2017 Aug 31;12(8):e0184294. doi: 10.1371/journal.pone.0184294 (PMC5578674; doi:10.1371/journal.pone.0184294)
Supplement: S1 Table — (DOCX) [file pone.0184294.s001.docx]

**Elevated aminopeptidase N affects sperm motility and early embryo development**

Amena Khatun^1¶^, Md Saidur Rahman^1¶^, Do-Yeal Ryu^1^, Woo-Sung Kwon^1^, and Myung-Geol Pang^1*^

S1 Table. Comparison of the average path velocity (VAP) and straightness (STR) between the control and APN-supplemented spermatozoa

|  | Average VAP (µm/s) | Average STR |
| --- | --- | --- |
| Control* | 61.72 | 84.76 |
|  | 59.38 | 84.55 |
|  | 57.03 | 81.78 |
|  | 58.01 | 82.64 |
| APN | 45.45 | 71.21 |
|  | 43.53 | 72.99 |
|  | 42.07 | 66.71 |
|  | 46.06 | 76.76 |

*Progressively motile spermatozoa > 50 µm/s VAP [30]

*Progressively motile spermatozoa ≥ 80 STR [31]
