# Supplementary material for: Elevated aminopeptidase N affects sperm motility and early embryo development
Source: PLoS One. 2017 Aug 31;12(8):e0184294. doi: 10.1371/journal.pone.0184294 (PMC5578674; doi:10.1371/journal.pone.0184294)

**Elevated aminopeptidase N affects sperm motility and early embryo development**

Amena Khatun^1¶^, Md Saidur Rahman^1¶^, Do-Yeal Ryu^1^, Woo-Sung Kwon^1^, and Myung-Geol Pang^1*^

**S1 Fig.** **Effect of different concentrations (2–200 ng/mL) of recombinant APN incubated for 30–90 min on the motility of mice spermatozoa.** Data represent the mean ± SEM of six replicates. **P* < 0.05, calculated using Tukey’s multiple comparison test.


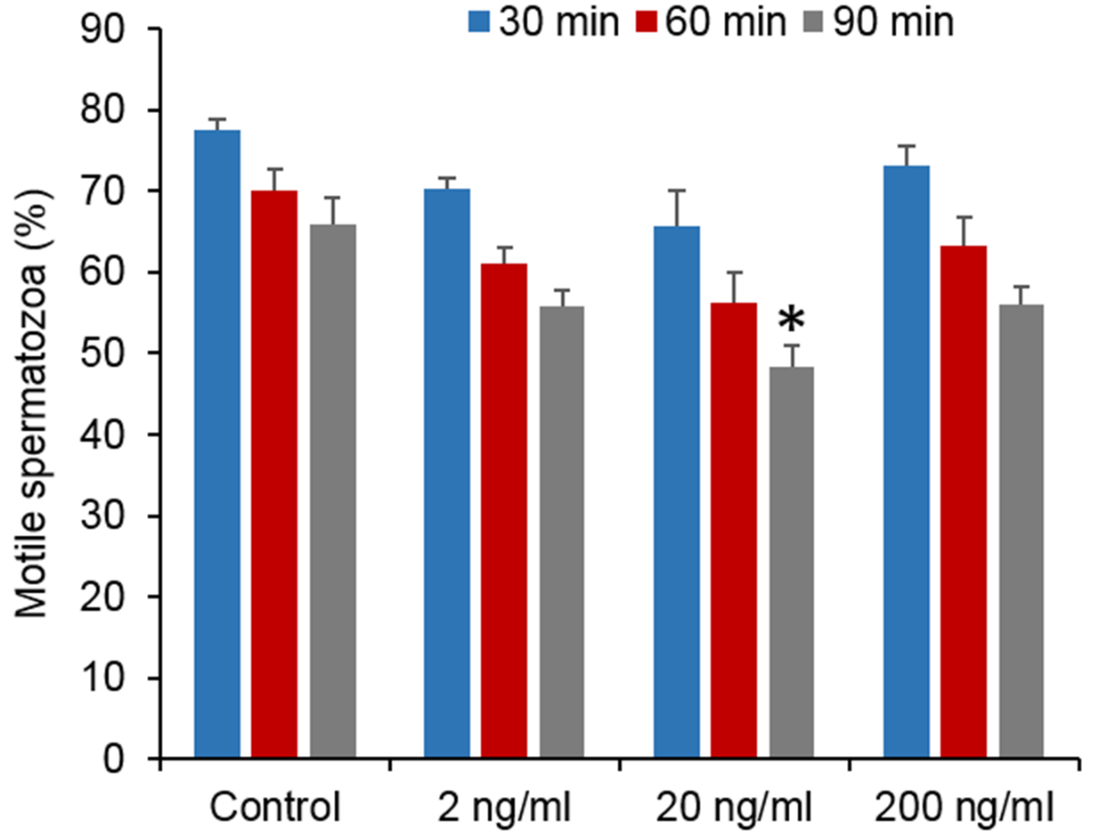

Supplement: S1 Fig — Data represent the mean ± SEM of six replicates. *P < 0.05, calculated using Tukey’s multiple comparison test. (DOCX) [file pone.0184294.s002.docx]
